# Physical Rehabilitation Subsequent to Fixation of Ilizarov Ring Fixator for the Management of Distal Femur Fracture: A Case Report

**DOI:** 10.7759/cureus.56201

**Published:** 2024-03-14

**Authors:** Ishwin Kaur B Bagga, Mitushi Deshmukh

**Affiliations:** 1 Musculoskeletal Physiotherapy, Ravi Nair Physiotherapy College, Datta Meghe Institute of Higher Education & Research, Wardha, IND

**Keywords:** case report, rehabilitation, strengthening, infection, ilizarov circular external fixator, chronic osteomyelitis, distal femur fracture

## Abstract

Distal femur fractures are severe all over the world. The goal of the study was to assess the effect of physiotherapy on ROM, strength, and improving quality of life. Due to the anatomy of distal femur fracture, the Ilizarov ring fixator is useful as it helps maintain mobility and stability. Distal femur fractures are most treated surgically compared to non-surgical treatment. The use of external fixators differs according to the patient’s condition and the stability of the patient. This study's objective was to evaluate the effectiveness of an evidence-based procedure prepared for the management of distal femur fracture and chronic osteomyelitis femur. In some cases, due to discharging sinus, the patient requires long-term treatment followed by a home physiotherapy rehabilitation program. The objective was to assess the effects of Ilizarov circular external fixators (ICEF) on distal femur fracture.

## Introduction

Fractures of the distal femur are serious. They occur at a frequency of about 0.4%, with different patterns in terms of who is affected. Typically, there are two peaks in occurrence: one among men in their 30s and another among elderly women [1,2]. Surgical reduction and stabilization of displaced, intra-articular distal femur fractures is usually recommended [1,3]. Only surgical therapy is indicated to fix the fracture due to the architecture [1]. External fixation, Ilizarov ring fixator, fixed-angle blade plates, etc., are all surgical fixation methods [4]. Non-union, whether septic or aseptic, is a frequent complication in open comminuted distal femur fractures. The broad approach and additional periosteal stripping involved in using a fixed-angle distal femur locking plate in these cases can potentially increase the risk of septic non-union [5,6]. Retrograde supracondylar nails are an excellent alternative since they have advantages such as low exposure and little blood loss [5,7,8]. These nails, however, are not appropriate for comminuted fractures of type C2 and C3 [5,9]. As a result, Ilizarov circular external fixators (ICEF) are employed [5]. Chronic osteomyelitis is a complicated bacterial infection with several characteristics in common [10].

Histopathologic features, rather than infection duration, are used to classify osteomyelitis as acute or chronic. Acute hematogenous osteomyelitis is caused by bacterial seeding of bone. Chronic osteomyelitis is usually caused by open fractures, bacteremia, or a contiguous soft tissue infection [11,12]. Open fractures or surgical treatment of closed injuries are the most prevalent causes of osteomyelitis. Patients often have a history of long-term disability and several surgical operations [13,14]. To have an understanding of the pathophysiology of osteomyelitis is necessary. In up to 80% of instances of osteomyelitis, Staphylococcus aureus is the causal pathogen [15-17]. Physiotherapy is beneficial in relieving post-operative pain and preventing complications. Physiotherapy regimens often comprise passive movement, active-aided movements, active movements, progressive resisted exercises to develop muscular strength, and cryotherapy for pain management [18-20].

## Case presentation

Patient information

A 37-year-old male resident of Morshi came to Acharya Vinobha Bhave Rural Hospital (AVBRH) with a history of slip and fall at home four months back sustaining an injury to the right femur with complaints of pain over a right limb and inability to bear weight on the right limb. Investigations like complete blood count and X-ray were done. The X-ray revealed a right distal femur fracture. The patient was suggested for operation and was thus managed with an Illizarov ring fixator over the right distal femur. Three months back he developed a discharging sinus post-operatively after the suture was removed from the right distal femur. After the necessary testing, the patient was diagnosed with chronic osteomyelitis of the right distal femur. After taking the pain history, the onset of pain was sudden and gradually progressive. It was more while moving the limb and was relieved with rest; nature was dull aching. It did not radiate to any other part of the body. For further management, he was recommended for physiotherapy.

Clinical findings

The patient's build was mesomorphic, and he was conscious and co-operative. The results of the physical examination were all normal. The patient's general assessment was normal, and he was vitally stable. The left-side lower limb had a full range of motion (ROM), and manual muscle testing (MMT) was also normal for the left side. There was a significant decrease in right-side strength and ROM. Table 1 shows the ROM of pre- and post-rehabilitation. Table 2 describes the grade MMT pre- and post-intervention.

**Table 1:** ROM assessment °symbol denotes the degree of ROM. ROM, range of motion

Joint	Right				Left	
	Pre-intervention		Post-intervention		Non-affected side	
	Active	Passive	Active	Passive	Active	Passive
Hip: flexion	0-90°	0-95°	0-110°	0-115°	0-110°	0-115°
Extension	0-5°	0-9°	0-15°	0-20°	0-20°	0-27°
Abduction	0-25°	0-30°	0-41°	0-45°	0-45°	0-45°
Adduction	0-20°	0-25°	0-30°	0-30°	0-30°	0-30°
Ankle: plantarflexion	0-18°	0-22°	0-34°	0-40°	0-45°	0-50°
Dorsiflexion	0-12°	0-15°	0-15°	0-20°	0-15°	0-20°

**Table 2:** MMT MMT, manual muscle testing

Muscle	MMT grade (right)		MMT grade (left)
	Pre-intervention	Post-intervention	Non-affected side
Hip: flexors	2	4	4
Extensors	2	4	4
Abductors	3	4	4
Adductors	3	4	4
Ankle: plantarflexors	3	4	4
Dorsiflexors	3	4	4

Investigations

**Figure 1:**
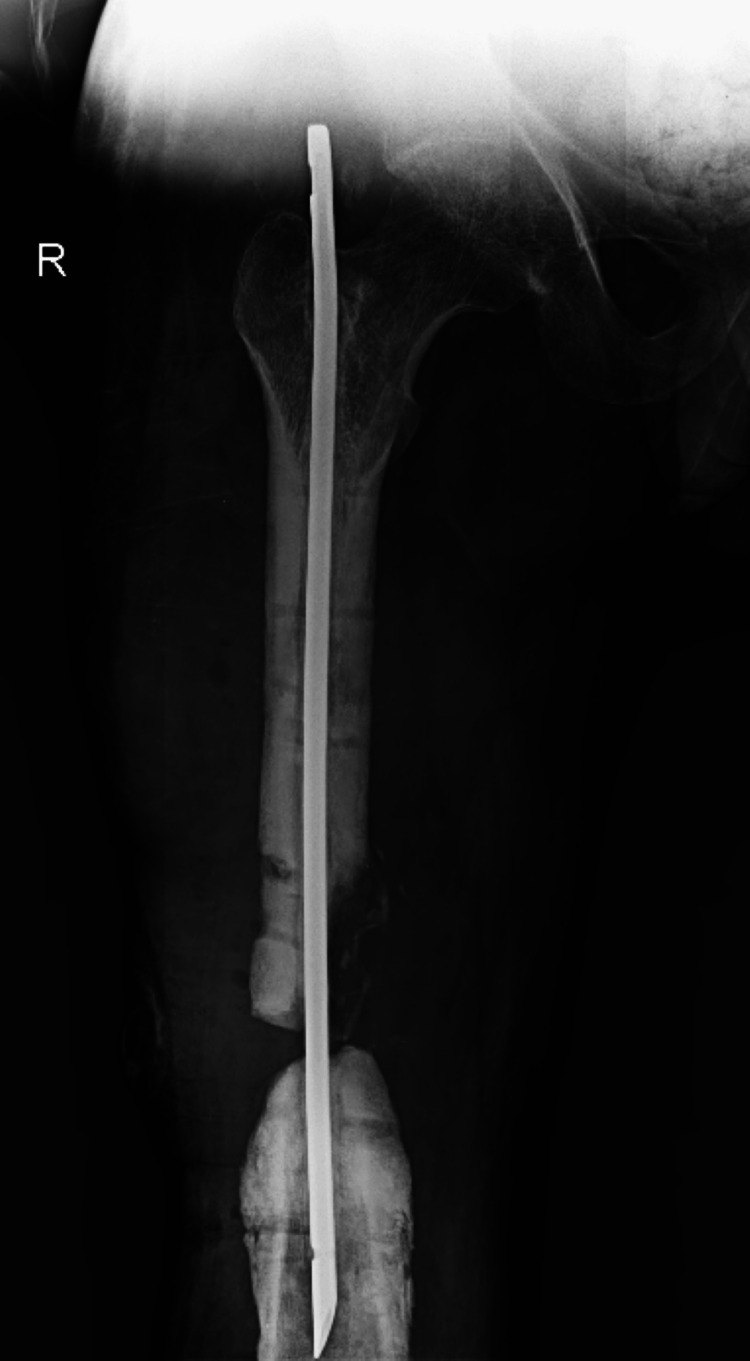
Early fracture X-ray

**Figure 2:**
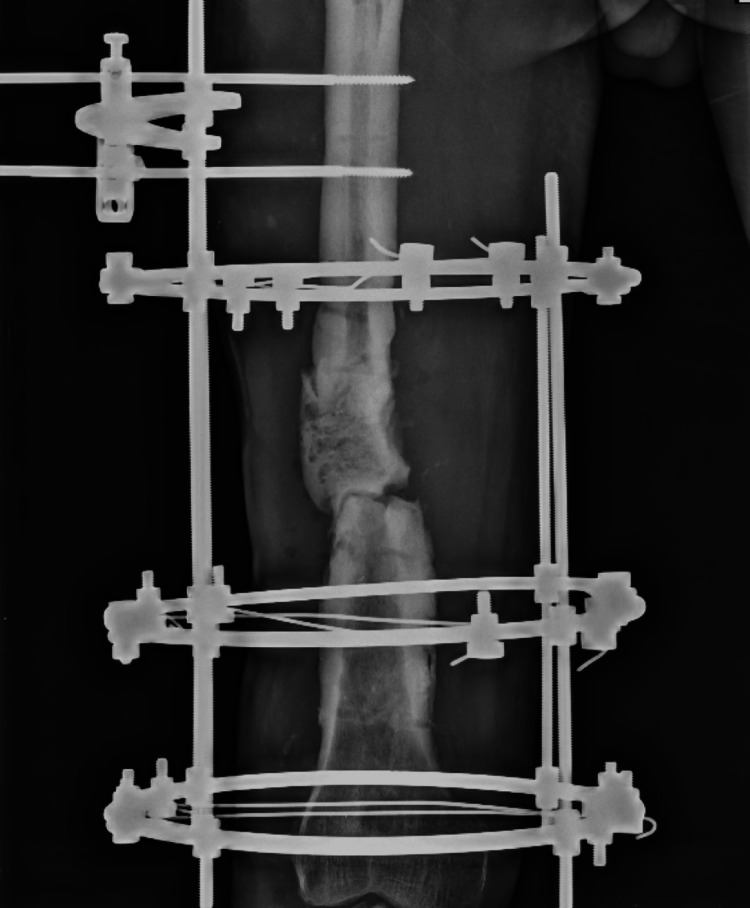
Ilizarov ring fixator X-ray

**Figure 3:**
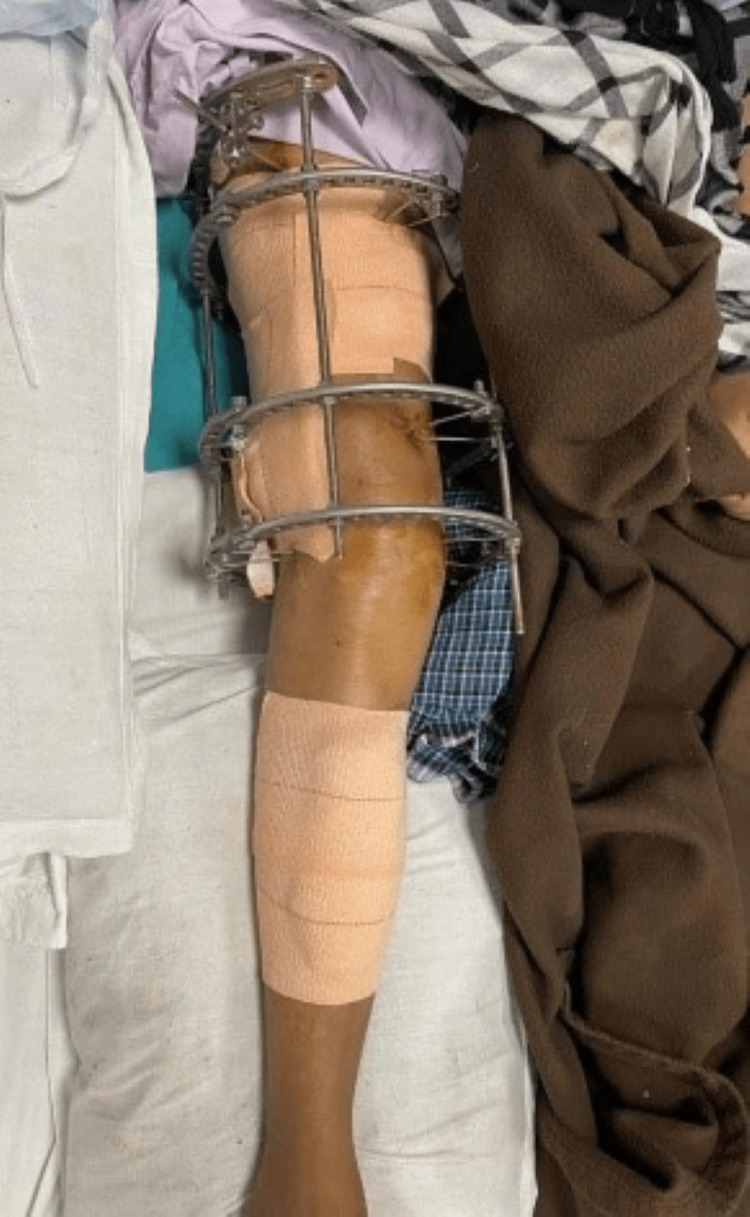
Post-operative stage

**Figure 4:**
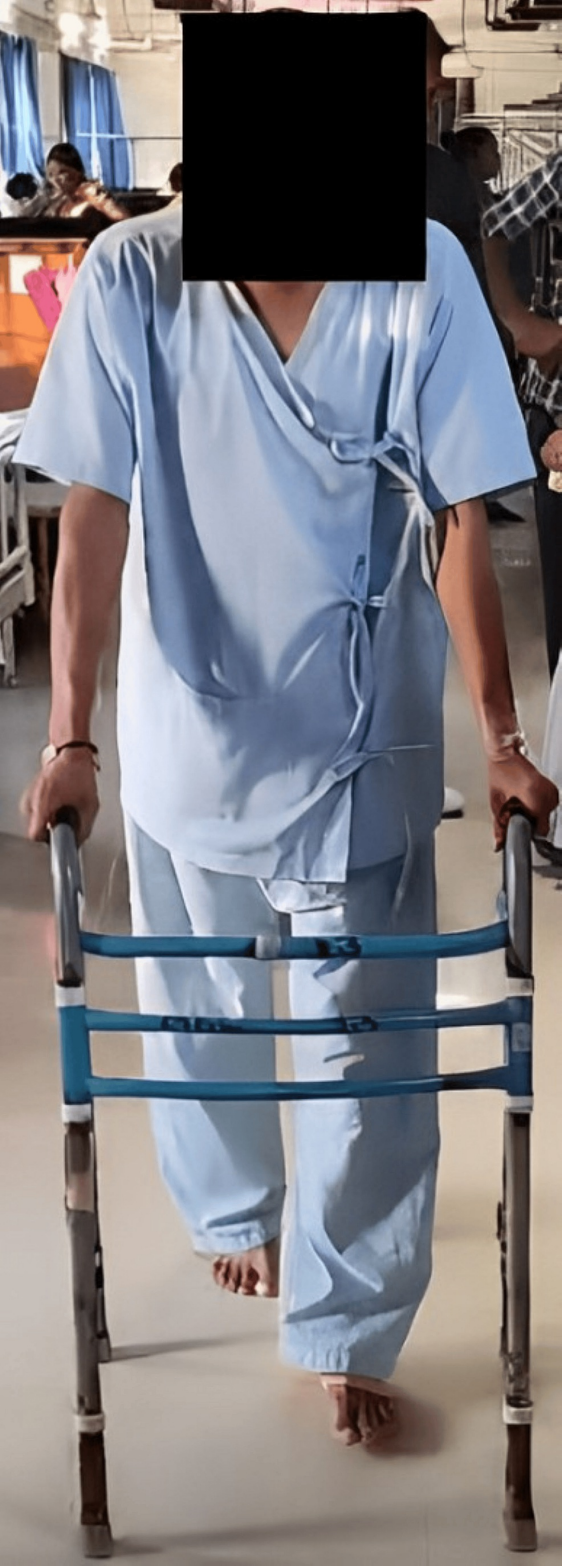
Ambulating the patient using a walker

The patient had an operated case of femur fracture and was surgically managed with the insertion of a rod before he came to the hospital with complaints. The patient's pre- and post-operative X-rays were taken and are mentioned below. Figure 1 shows the pre-Illizarov ring fixator X-ray. Figure 2 shows the post-Illizarov ring fixator X-ray. Figure 3 shows the post-operative stage of the patient. Figure 4 shows ambulating the patient using a walker after the removal of the Ilizarov ring fixator. 

Treatment

A planned physical therapy protocol was made for the patient. The patient had complaints of inability to stand independently and reduced strength. The goal of physiotherapy was to rebuild the patient's strength and restore his daily activities. Table 3 describes the treatment protocol, which was set for the patient.

**Table 3:** Treatment protocol MS, muscle strength; FA, functional ability; WB, weight-bearing; FS, fracture site; BH, bone healing, ROM, range of motion

Phase	Physiotherapy exercises
Phase 1: immediate post-operative phase (day 1 - week 1)	
Stability at FS	None
Stage of BH	Inflammatory phase
X-ray	No callus, fracture line clearly visible
Precaution	No passive ROM to hip and knee
To reduce pain and inflammation	Cryotherapy for 8-10 min, thermotherapy and ultrasound 2 times a day
ROM	Active ROM of hip and knee
MS	Isometric exercises to quads and hams, ankle pumps 10 reps, 1 set
FA	Ambulatory stand-pivot transfers
WB	Toe touch or non-WB
Phase 2: protection phase (week 2 - week 6)	
Stability at FS	None to minimal
Stage of BH	Beginning of reparative phase
X-ray	None to very carly callus
Precaution	Avoid rotation on right extremity
To improve endurance	Core stabilization exercises
ROM	Active, active-assisted ROM of hip and knee
MS	Isometric exercise to hams, quads, and glutes and straight leg raise, ankle pumps
FA	Ambulatory stand-pivot transfer using walker and ambulation with walker
WB	Partial WB to tolerable WB
Phase 3: Intermediate phase (week 6 - week 8)	
Stability at FS	With bridging callus, fracture is usually stable
Stage of BH	Reparative phase
X-ray	Bridging callus is visible
Precaution	Avoid rotation to excess ranges
To improve endurance	Modality - faradic current stimulation, continuous passive motion heel slides, ankle pumps
ROM	Active/passive ROM to hip and knee
MS	Resistive isotonic exercises and isometric exercises to hams, quads, and glutes
FA	Stand/pivot transfer and ambulation with walker
WB	Partial WB to full WB
Phase 4: advanced strengthening exercises (week 8 - week 10)	
Stability at FS	Stable
Stage of BH	Remodeling phase
X-ray	Abundant callus in fracture site
Precaution	Avoid torsion loading to femur
ROM	Active/passive ROM to hip and knee
MS	Progressive resistive exercises to quads, hams, and glutei
FA	Regular transfers and ambulation without walker
WB	Full WB

Outcome measures 

Visual analog scale, lower extremity functional scale (LEFS), and functional independence measure (FIM) were taken as outcome measures. The patient's progression was observed on the basis of progression of outcome measures. The visual analog scale was used to assess the pain, LEFS was used to assess the functional limitation, and the functional independence scale was used to assess the dependency of the patient. Table 4 shows the outcome measures used for the patient.

**Table 4:** Outcome measures used LEFS, lower extremity functional scale; FIM, functional independence measure

Outcome measure	Pre-intervention	Post-intervention
Visual analog scale	On rest: 6.7/10; on activity: 8.8/10 (severe pain)	On rest:2.1/10; on activity: 3.6/10 (mild pain)
LEFS	Score = 46 (moderate functional limitation)	Score = 72 (very minimal functional limitation)
FIM	Score = 32/126 (maximum dependency)	Score = 110/126 (functionally independent)

## Discussion

Distal femoral fracture treatment is a challenging, intra-articular, and comminuted condition that provides substantial complications [4]. It is found that physiotherapy rehabilitation can be beneficial in patients with osteomyelitis and the ones who underwent an operation for fracture [19]. Physiotherapy plays a crucial role in patients undergoing treatment with Ilizarov ring fixators, aiding in the restoration of mobility and function. It focuses on goals such as maintaining joint ROM, preventing muscle atrophy, and promoting optimal healing around the fixator site. By implementing targeted exercises and interventions, physiotherapists strive to optimize recovery, minimize complications, and enhance the overall quality of life for these patients. In this case study, we are talking about an instance of a 37-year-old male with a distal femur fracture treated with an Ilizarov ring fixator. In this instance, the main objectives of the physical intervention were to inform the patient and avoid deformities and other unintended consequences. Ankle pumps and heel slides were initiated to prevent secondary complications as they help in peripheral circulation enhancement. To increase the strength of the muscle’s static and dynamic hamstring, quadriceps and gluteal muscle strengthening were initiated. After the development of required strength, gait training using a walker was initiated, which was at first half weight-bearing and then progressed to full weight-bearing. Proper strengthening to both affected and unaffected limbs was given.

## Conclusions

In conclusion, this case report underscores the crucial role of physiotherapy in the successful rehabilitation following the fixation of distal femur fractures using the ICEF. Through meticulous attention to patient-specific needs, physiotherapy facilitates optimal healing, functional recovery, and restoration of mobility. By addressing muscle weakness, joint stiffness, and proprioceptive deficits, physiotherapy not only enhances the patient's physical capabilities but also promotes psychological well-being, enabling individuals to regain independence and quality of life post-injury. Emphasizing the significance of a multidisciplinary approach, this case highlights the synergy between surgical intervention and tailored rehabilitation strategies in achieving favorable outcomes for patients undergoing complex orthopedic procedures.
